# Overweight and Obesity and Associated Factors among School-Aged Adolescents in Ghana and Uganda

**DOI:** 10.3390/ijerph8103859

**Published:** 2011-09-28

**Authors:** Karl Peltzer, Supa Pengpid

**Affiliations:** 1 HIV/AIDS/STI/and TB (HAST), Human Sciences Research Council, 134 Pretorius Street, Pretoria 0002, South Africa; 2 Department of Psychology, University of Limpopo, P/B X1106, Sovenga 0727, South Africa; 3 Department of Health System Management and Policy, University of Limpopo, PO Box 197, Medunsa 0204, South Africa; E-Mail: supa_pengpid@embanet.com

**Keywords:** overweight, obesity, global school-based health survey, dietary behavior, physical activity, sedentary behavior, psychosocial factors, Ghana, Uganda

## Abstract

The aim of this study was to assess overweight and obesity and associated factors in school-going adolescents in low income African countries (Ghana, Uganda). The total sample included 5,613 school children aged 13 to 15 years from nationally representative samples from two African countries. Bivariate and multivariable analyses were conducted to assess the relationship between dietary behavior, substance use, physical activity, psychosocial factors and overweight or obesity. The prevalence of overweight and obesity was determined based on self-reported height and weight and the international child body mass index standards. Results indicate a prevalence of overweight or obesity of 10.4% among girls and 3.2% among boys, and 0.9% and 0.5% obesity only among girls and boys, respectively. Among girls smoking cigarettes and loneliness and among boys smoking cigarettes were found to be associated with overweight or obesity in multivariable analysis. Overweight status was not associated with the intake of fruits, vegetables, and sedentary behavior. Low prevalence rates of overweight or obesity were found in Ghana and Uganda. Smoking cessation and social programs could be integrated into strategies to prevent and treat overweight and obesity in youth.

## 1. Introduction

During the past two decades, the prevalence of overweight and obesity in children has increased worldwide [1]. Obesity in childhood and adolescence has adverse consequences on premature mortality and physical morbidity in adulthood [2] and is associated with impaired health during childhood itself. Once obesity is established in children (as in adults) it is hard to reverse [1]. Monitoring the prevalence of obesity in order to plan services for the provision of care and to access the impact of policy initiatives is essential [1].

The prevalence of overweight and obesity continues to remain low in many lower income countries [3], but it seems to be changing in some middle income countries. For example, the prevalence of obesity among South African 3 to 16 years old children was found to be 3.2% for boys and 4.9% for girls, and overweight 14% for boys and 17.9% for girls [4], another study found 7.8% of school children aged 10 to 15 years were overweight or obese [5], and yet a more recent study in rural South Africa found combined overweight and obesity was higher in girls (15 %) than boys (4 %), as was central obesity (15% and 2%, respectively) [6].

Factors associated with childhood overweight or obesity include lower physical activity levels [7,8], higher sedentary behaviour (such as television viewing times) [7,8], dietary behaviour such as frequency of sweets intake [8], psychosocial factors [9,10], female gender [6], victims and perpetrators of bullying behaviours [11], inaccurate perceptions of the need to diet, poorer self-perceived health status and potential social isolation [12]. Overweight status was not associated with the intake of fruits, vegetables [8,13]. Risk factors such as dietary behaviour, life style factors (smoking and alcohol use), physical activity and psychosocial factors for overweight in low-income countries are not well-known and might differ from those in other countries. Therefore, the aim of this study was to assess overweight and obesity and associated factors in school-going adolescents in African low income countries (Ghana, Uganda).

## 2. Methods

### 2.1. Description of Survey and Study Population

This study involved secondary analysis of existing data from the Global School-Based Health Survey (GSHS) from two African countries (Ghana 2007 and Uganda 2003). All African countries from which GSHS datasets with Body Mass Index (BMI) information were publicly available were included in this secondary analysis. Details and data of the GSHS can be accessed at http://www.who.int/chp/gshs/methodology/en/index.html. The aim of the GSHS is to collect data primarily from students of age 13–15 years. A two-stage cluster sample design was used to collect data to represent all students in grades 6, 7, 8, 9, and 10 in the country. At the first stage of sampling, schools were selected with probability proportional to their reported enrollment size. In the second stage, classes in the selected schools were randomly selected and all students in selected classes were eligible to participate irrespective of their actual ages. Students self-completed the questionnaires to record their responses to each question on a computer scan able answer sheet. The GSHS 10 core questionnaire modules address the leading causes of morbidity and mortality among children and adults worldwide: tobacco, alcohol and other drug use; dietary behaviors; hygiene; mental health; physical activity; sexual behaviors that contribute to HIV infection, other sexually transmitted infections, and unintended pregnancy; unintentional injuries and violence; protective factors and respondent demographics [14].

### 2.2. Measures

#### 2.2.1. Body Mass Index (BMI) Measurement and Overweight Classification

Height and body weight were based on self-reports. BMI was calculated as weight/height^2^ (kg/m^2^). The international age- and gender-specific child BMI cut-points were used to define overweight and obesity [15]. These cut-points were derived in a large international sample using regression techniques by passing a line through the health-related adult cut-points at 18 years. Youth with BMI values corresponding to an adult BMI of <25.0 kg/m^2^ were classified as normal weight and youth with BMI values corresponding to an adult BMI of L25.0 kg/m^2^ were classified as overweight. Thus, in this study overweight youth included those who were obese. The overweight youth were further subdivided into pre-obese (BMI corresponding to adult value of 25.0–29.9 kg/m^2^) and obese (BMI corresponding to adult value of L30.0 kg/m^2^) groups. The response rate on the BMI was for Uganda 94.4% and Ghana 95.2%.

#### 2.2.2. Fruits and Vegetables Consumption and Hunger

Fruits: “During the past 30 days, how many times per day did you usually eat fruit, such as ‘country specific examples’?” Response options were 1 = I did not eat fruit during the past 30 days, 2 = less than one time per day, 3 = 1 time per day to 7 = 5 or more times per day.

Vegetables: “During the past 30 days, how many times per day did you usually eat vegetables, such as ‘country specific examples’?” Response options were 1 = I did not eat vegetables during the past 30 days, 2 = less than one time per day, 3 = 1 time per day to 7 = 5 or more times per day. Adolescents indicated that they were consuming fruits (or vegetables) less than once a day was coded as having inadequate consumption patterns. The inadequate fruits and vegetables consumption variables were re-coded separately into two categories: inadequate fruits consumption (less than once = 1) and adequate fruits consumption (once or more a day = 0) and inadequate vegetable consumption (less than once = 1) and adequate vegetable consumption (once or more a day = 0).

Hunger: A measure of hunger was derived from a question reporting the frequency that a young person went hungry because there was not enough food at home in the past 30 days (response options were from 1 = never to 5 = always) (coded 1 = most of the time or always and 0 = never, rarely or sometimes).

#### 2.2.3. Substance Use

Smoking cigarettes was assessed with the question, “During the past 30 days, on how many days did you smoke cigarettes?” Response options included 1 = 0 days to 7 = all 30 days. Alcohol use was assessed with the question, “During the past 30 days, on how many days did you have at least one drink containing alcohol?” Response options included 1 = 0 days to 7 = all 30 days.

#### 2.2.4. Physical Activity

Leisure time physical activity was assessed by asking participants: “During the past 7 days, on how many days were you physically active for a total of at least 60 minutes per day?” and “During a typical or usual week, on how many days are you physically active for a total of at least 60 minutes per day?” Physical activity was defined as any activity that increases heart rate and makes one get out of breath some of the time. Physical activity can be done in sports, playing with friends, or walking to school. Some examples of physical activity are running, fast walking, biking, dancing, and football. Physical education or gym classes were not supposed to be included. According to the scoring protocol of the PACE + Adolescent Physical Activity

Measure and existing guidelines, physical activity were defined as obtaining at least 60 min of physical activity per day on at least five days per week. For analysis, the number of active days “during the past week” and the number of active days “during a typical week” were averaged.

Leisure time sedentary behavior was assessed by asking participants about the time they spend mostly sitting when not in school or doing homework: “How much time do you spend during a typical or usual day sitting and watching television, playing computer games, talking with friends, or doing other sitting activities” (3 hours of more per day).

#### 2.2.5. Psychosocial Distress Variables

Loneliness “During the past 12 months, how often have you felt lonely?” (Response options were from 1 = never to 5 = always) (Coded 1 = most of the time or always and 0 = never, rarely or sometimes).

Anxiety or worried During the past 12 months, how often have you been so worried about something that you could not sleep at night? (Response options were from 1 = never to 5 = always) (Coded 1 = most of the time or always and 0 = never, rarely or sometimes).

Sadness During the past 12 months, did you ever feel so sad or hopeless almost every day for 2 weeks or more in a row that you stopped doing your usual activities? (Response option 1 = yes and 2 = no) (Coded 1 = 1, 2 = 0).

Suicide plan “During the past 12 months, did you make a plan about how you would attempt suicide?” (Response option was 1 = yes and 2 = no, coded 1 = 1, 2 = 0).

No close friends “How many close friends do you have?” (Response options 1 = 0 to 4 = 3 or more, coded 1 = 1, 2–4 = 0.).

Bullied The variable “ever being bullied” was defined as those who reported they were bullied at least once in the preceding 30 days, by any form of bullying.

#### 2.2.6. Data Analysis

In order to compare study samples across countries each country sample was restricted to the age group 13–15 years, younger and older participants were excluded from the analyses. Data analysis was performed using STATA software version 10.0 (Stata Corporation, College Station, TX, USA). This software has the advantage of directly including robust standard errors that account for the sampling design, *i.e.*, cluster sampling owing to the sampling of school classes. In further analysis, the inadequate fruits and vegetables consumption variables were re-coded separately into two categories: inadequate fruits and vegetables consumption (less than once = 1) and adequate fruits and vegetables consumption (once or more a day = 0). Associations between dietary behavior and substance use, physical activity and psychosocial distress and overweight or obesity among school children were evaluated calculating odds ratios (OR). Unconditional logistic regression was used for evaluation of the impact of explanatory variables for overweight or obesity for boys and girls (binary dependent variables). All variables statistically significant at the P < 0.05 level in bivariate analyses were included in the multivariable models. In the analysis, weighted percentages are reported. The reported sample size refers to the sample that was asked the target question. The two-sided 95% confidence intervals are reported. The P values less or equal to 5% is used to indicate statistical significance. Both the reported 95% confidence intervals and the P value are adjusted for the multi-stage stratified cluster sample design of the study.

## 3. Results

### 3.1. Sample Characteristics

The study response rate for Ghana was 83% and for Uganda 69%. The study found a prevalence of overweight or obesity of 10.4% among girls and 3.2% among boys, and 0.9% and 0.5% obesity only among girls and boys, respectively. In terms of dietary behavior, most girls as opposed to boys had fruits or vegetables less than once a day, and 17% indicated that mostly or always they felt hungry. More than three quarters of students indicated physical inactivity, almost half that they would not walk to school and more than one quarter engaged in three or more hours sedentary behavior per day. Regarding psychosocial factors, being bullied was the most frequent one, followed by sadness, having a suicide plan and having no close friends; females scored significantly higher than boys on “no close friends”, “suicide plan” and “anxiety” (see Table 1).

### 3.2. Association with Overweight or Obesity

Among girls smoking cigarettes and loneliness was associated with overweight or obesity in bivariate and multivariable analysis, and among boys inadequate intake of fruits and smoking cigarettes were associated with overweight or obesity in bivariate analysis and smoking cigarettes were retained in multivariate analysis. Other dietary behaviour (eating vegetables, hunger), alcohol use, physical activity and other psychosocial factors (anxiety, sadness, suicide plan, no close friends, being bullied) were not found to be associated with overweight or obesity in girls or boys (see Table 2).

## 4. Discussion

The study found a prevalence of overweight or obesity of 10.4% among school-going girls and 3.2% among boys, and 0.9% and 0.5% obesity only among girls and boys, respectively, in Ghana and Uganda. These prevalence rates of overweight or obesity seem similar or lower than findings from middle income countries [4–6,16], and much lower than studies from high income countries (among 13-year-olds 10% in girls and 16% in boys and among 15-year-olds 10% in girls and 17% in boys) [8,17,18]. This study found a higher prevalence rate of overweight or obesity in female than male adolescents, which concurs with studies among adolescents in low and middle income countries [e.g., 4,6,19] but the opposite hold true in high income countries where adolescent boys had higher rates of overweight or obesity than girls [17,18]. Possible reasons for this may be cultural differences in beliefs regarding body image. In a study among Black African adolescent girls it was found that two thirds of the girls perceived fatness as a sign of happiness and wealth. Socially, fatness was accepted but one third of the girls had contradictory views about its advantages. Among obese girls who believed that being obese was preferable, the dominant reasons were that being fat allowed one to engage in sport activities that need strength and also makes one look respectable [20].

We studied health-risk behaviours that could influence energy metabolism such as alcohol and tobacco use [21]. Substance use (smoking but not alcohol use) was in this study significantly associated with overweight or obesity. In a review of 19 studies Potter *et al*. [22] found some evidence of a positive relationship between smoking and body weight among adolescents [23,24] but other studies did not find a positive association [16,21]. In a study from low and middle income countries Salazar-Martinez *et al*. [25] found among adolescents (11 to 19 years) from Mexico and Egypt that ever smoked was inversely associated with BMI and occasional smoker/ex-smoker was not associated with BMI. There may be a belief that adolescents, particularly girls, use smoking as a means to control body weight [22]. In a review Potter *et al*. [22] found “in terms of the relationship between weight concerns and adolescent smoking, the amount of evidence supporting a positive association differed depending on the dimension of weight concern considered, with the strongest evidence for dieting behaviours.” The findings in the literature on alcohol use and overweight among adolescents seem to be inconsistent, a few European studies [24,26] report a positive association and other studies [21], as in this study, no association between alcohol use and overweight was found. This study found similar levels of alcohol consumption by girls and boys, this concurs with findings from other African countries where 13 to 15 year-old boys and girls had a similar prevalence of risky drinking [27,28]. While prevalence rates for alcohol use and related disorders differ widely between adult men and women, male and female adolescents do not exhibit the same disparity in alcohol consumption [29]. For instance, recent (2010) data from the USA indicate that female and male adolescents (12–17 years) report similar rates for current drinking, 13.5% and 13.7% respectively [30].

In this study among girls a psychosocial factor (loneliness) was significantly associated with overweight or obesity. Fonseca and Gaspar de Matos also found that potential social isolation was associated with overweight among Portuguese adolescents [12]. Goodman and Whitaker [31] found in a longitudinal study that depressed adolescents are at increased risk for the development and persistence of obesity during adolescence. The importance of linking psychosocial stressors and childhood obesity has been stressed [32].

The effect of physical inactivity and sedentary behaviour was also explored in this study. Our study shows that the majority of adolescents were physical inactive; this lack of variability in physical activity might be due to measurement or real finding. In this case the lack of variability in the level of physical activity might account for the fact that none of physical activity and sedentary behaviour variables predicted overweight or obesity, as found in a few other studies [16] but which is contrary to most other studies [7,33]. Overweight status was in this study also not associated with the intake of fruits, vegetables, as found in other studies [8–13]. Further, the study found that being most of the time or always hungry was not associated with overweight. In a review of studies on food insecurity related to overweight and obesity in children and adolescents in the USA, Eisenmann *et al*. [34] found no associations between food insecurity and overweight among more recent studies with larger samples and that food insecurity and overweight co-exist.

## 5. Study Limitations

The study survey was cross-sectional and therefore no causal inferences can be made. The cut-offs used with self-reported BMI may lead to underestimation or overweight and obesity [35]. The BMI was assessed by self-reported weight and height and could have included anthropometry to evaluate weight status and body fat content [5]. In addition, a number of factors known to be associated with weight status were not assessed including dietary intake, low quality diet, skipping breakfast, environmental, family variables including parental weight status, socioeconomic status [9,16,36,37], age at menarche and order of birth [13,16].

## 6. Conclusions

Low prevalence rates of overweight or obesity were found among adolescents in Ghana and Uganda. Smoking cessation and social programs could be integrated into strategies to prevent and treat overweight and obesity in youth.

## Figures and Tables

**Table 1:** Descriptive data of school-going adolescents aged 13–15 years (N = 5,613).

	Male (n = 2,738) % [95% CI]	Female (n = 2,875) % [95% CI]
*Weight*		
Overweight or Obese	3.2 [2.4–4.1]	10.4 [8.8–12.1]
Obese	0.5 [0.2–0.9]	0.9 [0.4–1.4]
*Dietary behaviour and substance use*		
Fruits less than once a day	28.9 [26.7–31.2]	73.4 [70.5–76.4]
Vegetables less than once a day	28.9 [25.5–32.2]	71.6 [68.4–74.9]
Most of the time or always hunger	18.4 [15.7–21.1]	16.4 [14.9–18.0]
*Days of alcohol use in past month*		
1 or 2 days	10.4 [8.5–12.3]	11.8 [9.9–13.7]
3 to 5 days	5.0 [3.8–6.2]	5.0 [3.6–6.4]
6 to 9 days	2.4 [1.5–3.3]	2.0 [1.1–3.0]
10 to 19 days	1.7 [1.1–2.2]	1.4 [0.8–2.1]
20 to 29 days	0.8 [0.2–1.5]	0.7 [0.4–1.1]
All 30 days	2.2 [1.4–3.0]	2.0 [1.1–2.9]
*Days of smoking in past month*		
1 or 2 days	3.7 [2.4–5.0]	1.1 [0.4–1.8]
3 to 5 days	1.1 [0.3–1.9]	0.8 [0.2–1.6]
6 to 9 days	0.5 [0.0–0.9]	0.1 [0.0–0.6]
10 to 19 days	0.5 [0.1–1.2]	0.4 [0.0–0.8]
20 to 29 days	0.2 [0.0–0.7]	---
All 30 days	0.2 [0.0–0.9]	0.2 [0.0–0.6]
*Physical activity*		
Physical activity less than 60 min per day on at least five days per week	78.5 [73.7–83.0]	84.9 [82.8–87.1]
Not walk or bike to school at least once a week	49.1 [45.1–53.0]	43.3 [39.8–46.8]
Sedentary behavior (3 hours of more per day)	27.1 [23.6–30.5]	26.9 [24.3–29.6]
*Psychosocial factors*		
Loneliness	8.5 [6.3–10.7]	11.2 [9.3–13.0]
Anxiety or worried	6.5 [4.6–8.4]	11.0 [8.7–13.3]
Sadness	36.9 [33.1–40.6]	40.9 [36.9–44.9]
Suicide plan	14.7 [11.9–17.5]	24.3 [19.6–29.0]
No close friends	7.8 [5.7–9.8]	19.1 [15.5–22.6]
Being bullied	45.7 [41.8–49.6]	51.5 [47.9–55.1]

**Table 2:** Overweight or obesity regression model.

	Female		Male	
	OR^1^	AOR^2^	OR	AOR
*Dietary behaviour and substance use*				
Fruits less than once a day	0.91 (0.66–1.26)	—	1.53 (1.02–2.28)^*^	1.34 (0.46–3.86)
Vegetables less than once a day	1.02 (0.78–1.35)	—	1.20 (0.86–1.67)	—
Most of the time or always hunger	0.91 (0.65–1.26)	—	0.84 (0.37–1.91)	—
Days of alcohol use in past month	0.92 (0.80–1.05)	—	0.98 (0.83–1.15)	—
Days of smoking in past month	1.68 (1.22–2.30)^**^	1.52 (1.17–1.98)^**^	1.76 (1.12–2.73)^*^	1.75 (1.11–2.76)^*^
*Physical activity*				
Physical activity less than 60 min per day on at least five days per week	0.80 (0.53–1.19)	—	0.59 (0.35–1.02)	—
Not walk or bike to school at least once a week	1.02 (0.76–1.25)	—	1.43 (0.84–2.45)	—
Sedentary behavior (3 hours of more per day)	1.01 (0.67–1.51)	—	1.33 (0.75–2.35)	—
*Psychosocial factors*				
Loneliness	2.24 (1.23–4.07)^**^	2.26 (1.23–4.15)^*^	0.80 (0.10–6.55)	—
Anxiety or worried	1.15 (0.63–2.12)	—	1.96 (0.55–7.01)	—
Sadness	1.13 (0.78–1.64)	—	2.38 (0.89–6.39)	—
Suicide plan	1.41 (0.73–2.72)	—	3.56 (0.83–15.32)	—
No close friends	1.25 (0.74–2.12)	—	0.36 (0.04–3.46)	—
Being bullied	0.90 (0.65–1.24)	—	1.04 (0.66–1.64)	—

1 OR = Odds Ratio;

2 AOR = Adjusted Odds Ratio.
